# Mortality among extrapulmonary tuberculosis patients in the HIV endemic setting: lessons from a tertiary level hospital in Mbeya, Tanzania

**DOI:** 10.1038/s41598-024-61589-z

**Published:** 2024-05-13

**Authors:** Erlend Grønningen, Marywinnie Nanyaro, Bjørn Blomberg, Shoaib Hassan, Esther Ngadaya, Tehmina Mustafa

**Affiliations:** 1 Department of Global Public Health and Primary Care, Centre for International Health, University of Bergen, 5020 Bergen, Norway; 2 Department of Thoracic Medicine, Haukeland University Hospital, 5021 Bergen, Norway; 3 National Institute for Medical Research, Muhimbili Medical Research Centre, Dar es Salaam, United Republic of Tanzania; 4 Department of Clinical Science, Faculty of Medicine, University of Bergen, 5020 Bergen, Norway; 5 Department of Medicine, National Center for Tropical Infectious Diseases, Haukeland University Hospital, 5021 Bergen, Norway

**Keywords:** Outcomes research, HIV infections, Tuberculosis, Risk factors

## Abstract

Extrapulmonary tuberculosis (EPTB) has received less attention than pulmonary tuberculosis due to its non-contagious nature. EPTB can affect any organ and is more prevalent in people living with HIV. Low- and middle-income countries are now facing the double burden of non-communicable diseases (NCDs) and HIV, complicating the management of patients with symptoms that could be compatible with both EPTB and NCDs. Little is known about the risk of death of patients presenting with symptoms compatible with EPTB. We included patients with a clinical suspicion of EPTB from a tertiary level hospital in Mbeya, Tanzania, to assess their risk of dying. A total of 113 (61%) patients were classified as having EPTB, and 72 (39%) as having non-TB, with corresponding mortality rates of 40% and 41%. Associated factors for mortality in the TB groups was hospitalization and male sex. Risk factors for hospitalization was having disease manifestation at any site other than lymph nodes, and comorbidities. Our results imply that NCDs serve as significant comorbidities amplifying the mortality risk in EPTB. To strive towards universal health coverage, focus should be on building robust health systems that can tackle both infectious diseases, such as EPTB, and NCDs.

## Introduction

While *SARS-CoV-2* briefly surpassed tuberculosis (TB) as the leading infectious disease cause of death, the death toll of TB itself increased by 200,000 during the pandemic^1^. Best estimates are that 10.6 million fell ill with TB in 2021, causing around 1.6 million deaths. Of those who fell ill with TB, an estimated 7% had coinfection with Human Immunodeficiency Virus (HIV), and TB remains the leading cause of death among people living with HIV (PLHIV). Extrapulmonary tuberculosis (EPTB) has received lesser attention by the global public health programmes as compared to pulmonary TB (PTB) due to its non-contagious nature. However, EPTB contributed to 17% of notified global TB cases in 2021 and is associated with significant morbidity and mortality. EPTB is more prevalent in children and in PLHIV, and with reduced CD4 counts the proportion of EPTB relative to PTB increases^2,3^.

As the microbiological confirmation of EPTB is challenging, many patients are treated for EPTB on clinical suspicion in the low-resource setting^3,4^. The currently used diagnostic tools require invasive sampling, have reduced sensitivity in extrapulmonary samples and are underutilized^3–5^. This could potentially lead to both an over- and underestimation of EPTB and its contribution to the global burden of TB. At the patient level, poor diagnostics may result in wrongful or delayed diagnosis, late initiation of appropriate treatment, and subsequent increase in sequela and mortality.

EPTB is a heterogeneous disease and mortality rates differ according to organ manifestation^5^. The most common sites of infection are lymph nodes, pleura, bones and joints, abdomen, meninges, and the genitourinary tract^4^. The clinical consequences of EPTB vary greatly from the relative benign course of tuberculous lymphadenitis to severe neurological debilitation in tuberculous spondylitis, and very high mortality in tuberculous meningitis^6^. A HIV registry study found a similar mortality in EPTB and PTB of 11% vs 10%^4^. Most studies on the mortality rates in EPTB are registry studies^4,5,7^, mainly dealing with presumptive EPTB cases analyzed retrospectively. Therefore, mortality rates and mortality risk for a patient presenting with symptoms compatible with EPTB in the routine diagnostic setting remains a knowledge gap.

Tanzania is on the WHO list of 30 high-burden TB countries, was ranked number 24 in terms of incidence rate, and 14 in the world in terms of estimated number of cases^8^. The country also has an overlapping HIV epidemic. In 2021 there were 87,415 notified TB cases, of which 16,233 (19%) were extrapulmonary. Best estimate of incidence was 132,000 with 18,000 and 7800 deaths in HIV uninfected and PLHIV, respectively^1^. Of those tested for HIV, 18% were found to be HIV co-infected and 99% were on anti-retroviral therapy^1,9^. Mbeya Zonal Referral Hospital offers tertiary care services to approximately eight million people in six regions in the Southern Highlands of Tanzania. HIV prevalence in Mbeya region among adults (> 15 years) is 9%^10^.

In our previous study on the validation of a new immunochemistry-based antigen detection test (MPT64 test) for EPTB we have previously published mortality data from Mbeya in children. In our pediatric cohort we found a mortality rate of 23% in children (< 18 years old) with malnutrition as the main comorbidity^11^. The aims of this study were to assess the mortality rate and factors associated with mortality in adult (> 18 years old) patients presenting with symptoms compatible with EPTB at a tertiary level hospital in Tanzania.

## Materials and methods

**Figure 1:**
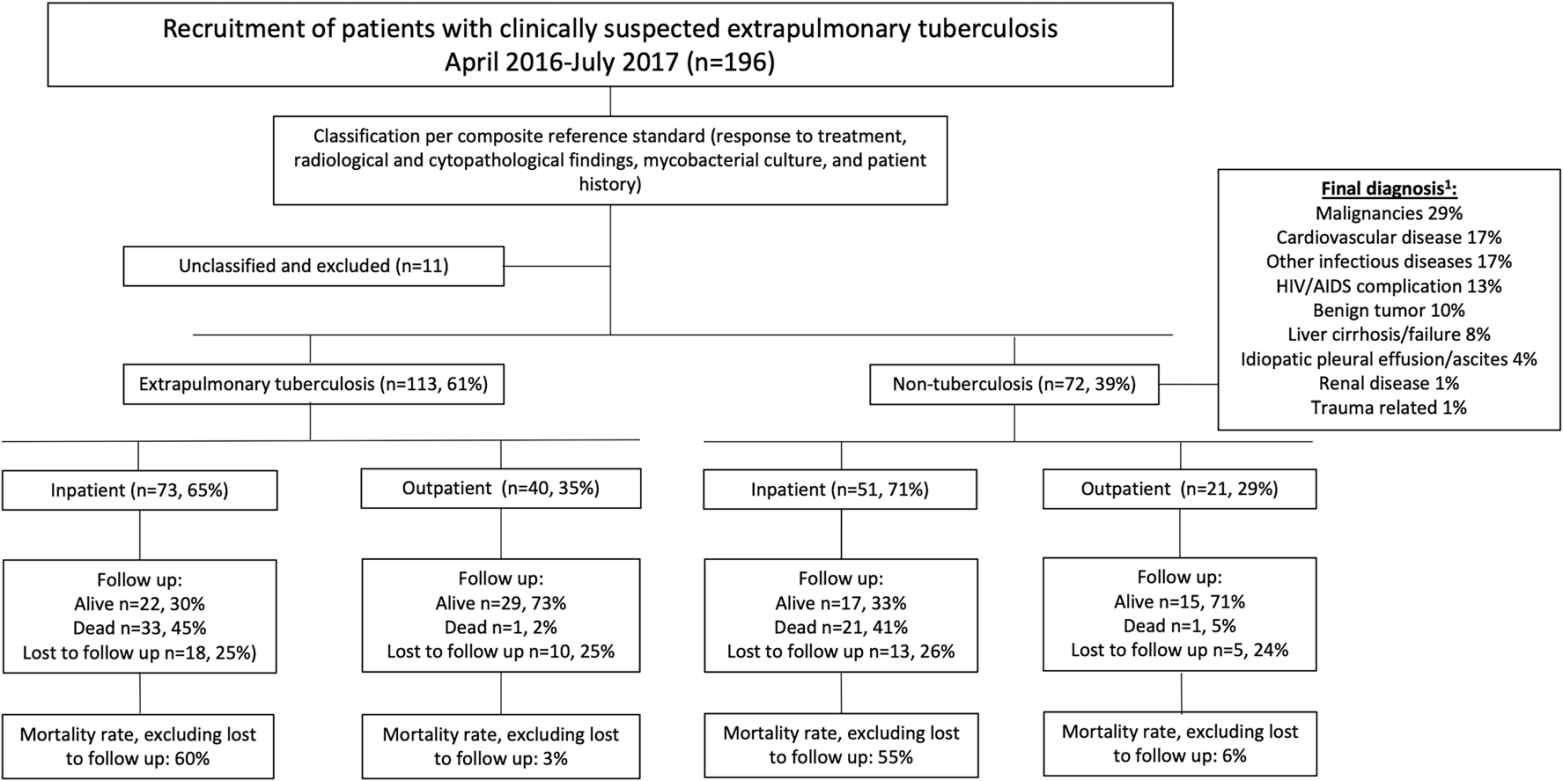
Inclusion, classification of patients and mortality rates according to department. ^1^Description of which diagnoses that are found under each heading can be found in the Supplementary Table S1.

This study was part of a prospective cohort study to validate the MPT64 test conducted at Mbeya Zonal Referral Hospital (MZRH) between April 2016 and July 2017^11^. All adult (> 18 years old) patients were nested to analyze the mortality rates and associated factors. As part of the diagnostic validation study, clinicians had been requested to include patients with presumed EPTB from both the in- and outpatient services. The inclusion of patients is shown in Fig. 1.

As part of the diagnostic validation study, patients were followed up at 2–3 months after initiation of anti-tuberculosis therapy (ATT), at the end of their ATT, or until an alternative diagnosis was established. As part of the study the researchers were granted access to the electronic hospital records at MZRH. Follow ups were arranged through medical officers hired for the purpose of the study. For the mortality analysis patients were followed for an additional six months after the last enrollments in July 2017. If patients were marked as lost to follow up a new tracing was done in 2018.

### Ethical considerations

The project was approved by the Ethical committee for biomedical research at the National Medical Research Coordinating Committee in Tanzania (NIMR/HQ/R.8a/Vol.IX/2142) and the regional Committee for Medical Research Ethics in Norway, REK Helse-Vest (2014/46/REK vest). HIV testing was performed per national guidelines, and not primarily for the study. To be included in the study all patients needed to provide their informed consent after having been given information about the study. All relevant national protocols were followed in the handling of patients.

### Classification of patients

The study participants were classified into TB and non-TB groups. The TB case definition relied on a composite reference standard^11,12^. A confirmed TB case was positive on mycobacterial culture. Probable TB was defined as clinically presumed EPTB and response to ATT at 2/3 months and/or end of treatment or clinically presumed EPTB with confirmed pulmonary TB and one of the following: radiological findings suggestive of EPTB, cytological findings compatible with TB in effusions/CSF and morphological features consistent with TB on fine needle aspiration cytology/biopsy. Possible TB was defined as a strong clinical suspicion from history/records and/or patient initiated ATT and one of the following: response to ATT at 2/3 months or end of treatment, radiological findings suggestive of EPTB, cytological findings compatible with TB in effusions/CSF and morphological features consistent with TB on fine needle aspiration cytology/biopsy. For this study, confirmed, probable and possible TB cases were merged to one category: TB. Patients for whom another conclusive diagnosis was reached and patients who did not meet the criteria of a TB case were labelled as non-TB. The grouping of final diagnoses of the non-TB patients is shown in Fig. 1.

Upon inclusion in the study the participants were asked to provide information about their prior medical history, medication, and HIV serostatus. Additional HIV testing was done on clinical request per national protocols, and not routinely^13^.

### Definitions

#### Status on follow up

Patients were marked as being alive if they responded to the phone follow-ups or had a continuous electronic trace in the hospital records. They were marked as dead if their passing was noted in the hospital records, or if the information was given on phone follow-up. Mortality in the TB group was defined as death by any cause.

#### Lost to follow up

Patients were marked as lost to follow up if they were unreachable by phone at 2–3 months and at the conclusion of ATT. If they responded to the additional tracing in 2018, the label was removed. The label was also removed if the electronic records in the hospital showed continuous examinations and follow up.

#### Response to treatment

Patients were scored on four parameters at 2–3 months and at the end of their ATT: improvement in symptoms as reported by the patient, improvement in symptoms evaluated by the clinician, weight gain and regression of objective findings. Each positive finding was given one point, and if the combined score was ≥ 3, the patients were marked as responding to treatment. The score at end of ATT was given priority over the score at 2–3 months.

#### Final non-TB diagnosis and comorbidities

Extensive information about additional comorbidities was found in the hospital records.

The diagnosis contributing the most to the patient’s illness was labelled as the final diagnosis by the first author, and other diseases were coded as comorbidities. No autopsies were performed. The comorbidities were grouped as shown in Table 1. Specific information about which conditions that were grouped together is found in the Supplementary Tables S1 and S2.

**Table 1:** Characteristics of patients that were either alive or dead on follow up.

	TB (n = 85)			Non-TB (n = 54)		
	Alive (n = 51, 60%)	Dies (n = 34, 40%)	OR (CI)	Alive (n = 32, 59%)	Dies (n = 22, 41%)	OR (CI)
Age (median–range)	32 (19–75)	41 (20–73)	1.04 (1.00–1.08)^1^	45 (22–85)	40 (20–74)	0.999 (0.96–1.04)^1^
Gender						
Male	24 (47%)	26 (77%)	3.66 (1.39–9.59)^2^	22 (69%)	14 (64%)	0.80 (0.25–2.50)^2^
Female	27 (53%)	8 (23%)	1	10 (31%)	8 (36%)	1
Department						
Inpatient	22 (43%)	33 (97%)	43.5 (5.52–343.1)^2^	17 (53%)	21 (95%)	18.5 (2.22–154.8)^2^
Outpatient	29 (57%)	1 (3%)	1	15 (47%)	1 (5%)	1
HIV status^3^						
HIV infected	28 (60%)	18 (58%)	0.87 (0.35–2.16)^2^	14 (45%)	11 (52%)	1.336 (0.44–4.06)^2^
HIV uninfected	19 (40%)	14 (42%)	1	17 (55%)	10 (48%)	1
Antiretroviral therapy coverage (% of HIV infected)						
On antiretroviral therapy	28 (100%)	16 (89%)	1	13 (93%)	10 (91%)	0.77 (0.04–13.86)^2^
Not on antiretroviral therapy	0 (0%)	2 (11%)	2.75 (1.86–4.07)^4^	1 (7%)	1 (9%)	1
Given anti-tuberculosis therapy						
No	7 (14%)	15 (44%)	4.96 (1.74–14.13)^2^	25 (78%)	17 (77%)	0.95 (0.26–3.50)^2^
Yes	44 (86%)	19 (46%)	1	7 (22%)	5 (23%)	1
Response to treatment	42/44 (95%)	–				
Suspected site of infection categorical						
Adenitis	29 (57%)	7 (21%)	1	12 (38%)	4 (18%)	1
All other TB forms	22 (43%)	27 (79%)	5.08 (1.87–13.81)^2^	20 (53%)	18 (82%)	2.70 (0.74–9.89)^2^
Pleuritis	10 (19%)	8 (24%)	3.31 (0.96–11.49)^2^	9 (28%)	3 (14%)	1.00 (0.18–5.63)^2^
Peritonitis	3 (6%)	7 (21%)	9.67 (1.98–47.14)^2^	6 (19%)	7 (32%)	3.500 (0.73–16.85)^2^
PTB with EPTB	4 (8%)	5 (14%)	5.18 (1.10–24.46)^2^	0	2 (9%)	NA
Multiorgan involvement^5^	4 (8%)	5 (14%)	5.18 (1.10–24.46)^2^	1 (3%)	6 (27%)	18.00 (1.63–198.5) ^2^
Mastitis	1 (2%)	0	NA	3 (9%)	0	NA
Other^6^	0	2 (6%)	NA	1 (3%)	0	NA
Main comorbidities (comorbidity present %)^7^						
No comorbidity	18 (35%)	5 (15%)	1	4 (13%)	1 (4%)	1
At least one comorbidity (including HIV)	33 (65%)	29 (85%)	3.16 (1.04–9.59)^2^	28 (87%)	21 (96%)	3.00 (0.31–28.84)^2^
Hematological diseases	5 (10%)	8 (23%)	2.83 (0.84–9.55)^8^	4 (13%)	9 (41%)	4.85 (1.26–18.68)^8^
Liver disease	1 (2%)	4 (12%)	6.67 (0.71–62.47)^8^	2 (6%)	7 (32%)	2.33 (1.36–4.01)^8^
Heavy alcohol consumption	0 (0%)	4 (12%)	NA	1 (3%)	2 (9%)	3.10 (0.26–36.48)^8^
Malignancy	1 (2%)	5 (15%)	8.62 (0.96–77.43)^8^	8 (25%)	8 (36%)	1.71 (0.53–5.59)^8^
Renal disease	1 (2%)	5 (15%)	8.62 (0.96–77.43)^8^	3 (9%)	3 (14%)	1.53 (0.28–8.37)^8^
Cardiovascular disease	4 (8%)	5 (15%)	2.03 (0.50–8.17)^8^	6 (19%)	8 (36%)	2.48 (0.72–8.57)^8^
Diabetes mellitus	0 (0%)	0 (0%)	NA	0 (0%)	1 (4%)	NA
Other infectious diseases	5 (10%)	7 (21%)	2.36 (0.69–8.26)^8^	12 (38%)	3 (14%)	0.26 (0.06–1.08)^8^
TB complication	2 (4%)	1 (3%)	0.74 (0.07–8.52)^8^	–	–	NA
HIV complication	3 (6%)	10 (31%)	7.65 (1.95–30.11)^8^	5 (16%)	6 (29%)	2.03 (0.53–7.72)^8^
Smoking	8 (16%)	8 (23%)	1.65 (0.55–4.94)^8^	9 (28%)	6 (27%)	0.96 (0.29–3.23)^8^

^1^Odds Ratio per year change in age.

^2^Logistic regression. Reference given value 1.

^3^Unknown HIV serostatus removed from analysis. 6 in TB group, 2 in non-TB group.

^4^Odds ratio for death if patient is not on ART.

^5^Includes disseminated TB, EPTB two sites and miliary TB.

^6^Includes cyst in abdomen (2) and scrotum.

^7^Description of which comorbidities found under each heading are found in the Supplementary Table S2.

^8^Logistic regression. Not having the comorbidity used as reference.

#### Department

Patients recruited from the inpatient department were marked as inpatients, and patients recruited from the outpatient departments as outpatients. If an outpatient was admitted to the hospital during the follow up period, the label was changed to inpatient.

### Statistical analysis

Statistical Package for Social Sciences, version 27.0.1.0 (IBM, Armonk, NY) was used for statistical analysis. Categorical variables were assessed using crosstabulation and odds ratios (OR) with 95% confidence intervals (CI) or Chi square test or Fishers exact test (when a cell count was < 5). The cutoff for statistical significance was defined by a p-value < 0.05. If the 95% CI for an odds did not include 1.0, the OR was considered as statistically significant at the 5% level. Logistic regression was used to analyze factors contributing to mortality.

## Results

### Mortality rates

The clinicians in MZRH included a total of 196 patients with clinically presumed EPTB that all gave consent to participate in this study, as shown in Fig. 1. After classification of the patients, 11 were unclassifiable and excluded, leaving 185 study participants, of which 113 (61%) were classified as having EPTB, and 72 (39%) as having non-TB. Malignancies (29%), cardiovascular diseases (17%) and other infectious diseases (17%) were the major non-TB diagnoses. In the TB group 54% were HIV infected, 39% HIV negative and 7% had an unknown HIV serostatus. In the non-TB group, the corresponding numbers were 46%, 50% and 4%, respectively.

After excluding patients that were lost to follow up from the analysis, we found an overall mortality rate of 40% in the 85 patients in the TB group and 41% in the 54 patients in the non-TB group. For the patients recruited as inpatients the corresponding mortality rates were 60% and 55%, respectively.

### TB groups vs non-TB group

The patients that died in the TB group were significantly older than those that survived (median 32 years vs 41 years, alive vs dead, respectively, OR 1.04 (CI 1.00–1.08)), and males contributed more to the mortality rate than females (77% vs 23%, respectively, OR 3.66 (CI 1.39–9.59)), as shown in Table 1. In both the TB and non-TB group, inpatients had much higher mortality rates, and only one patient from both groups died after being recruited from the outpatient department. There was no significant difference in HIV seropositivity among those who survived or died in either the TB or non-TB group.

Mycobacterial culture was positive in 22/85 (26%) TB cases.

In the non-TB group 12/54 (22%) were given ATT, but no significant difference was found in the mortality rates between those who received ATT and those who did not.

TB patients presenting with non-adenitis manifestation had a higher mortality rate than adenitis cases, contributing to 79% of cases that died (OR 5.08 (CI 1.87–13.81)), as shown in Table 1. The TB sites with the highest case fatality rates were peritonitis with 7/10 (70%), PTB with extrapulmonary involvement 5/9 (56%) and multiorgan involvement 5/9 (56%). All seven cases of TB adenitis who died had severe comorbidities.

Comorbidities were grouped into ‘no comorbidity’ and ‘any comorbidity’ present. The TB patients with any comorbidity had a statistically significant higher odds of dying (OR 3.16 (CI 1.03–9.59)). Among the TB patients who died 5 (15%) had no comorbidity and 29 (85%) had any comorbidity. In those who survived the corresponding numbers were 18 (35%) and 33 (65%).

Among the HIV infected in the TB group 33% (15/46) had current CD4 counts available (mean 185, range 31–360). In the non-TB group, the corresponding number was 28% (7/25, mean 279, range 86–415). There was no statistically significant difference in CD4 counts between the groups TB/non-TB or those that survived/died. Viral loads were not reported in the electronic hospital records and was therefore not available.

In the TB group, 13% (6/46 of the HIV infected) declared an unknown or negative HIV status on inclusion in the study but tested positive.

#### TB patients not receiving ATT

In the TB group, a total of 22/85 (26%) patients did not receive ATT. Among these 15/22 died, contributing to 44% of the mortality in the TB group (OR 4.96 (CI 1.74–14.13)), as shown in Table 1. The TB diagnosis was confirmed by our composite reference standard.

Among these 22 cases, four (18%) were confirmed TB cases and 18/22 (82%) were possible TB cases. Among the confirmed cases 75% (3/4) died and 67% (12/18) died in the possible TB group. Case fatality rates in the possible group was 40% (2/5) for pleuritis, 100% (5/5) for peritonitis, 57% (4/7) for adenitis, 67% (2/3) for PTB with EPTB, 100% (1/1) for multiorgan involvement and 100% (1/1) for other (cyst in abdomen).

### Inpatients vs outpatients

There was no difference in the age, gender, or HIV serostatus of patients recruited as inpatients compared to those recruited as outpatients in both groups, as shown in Table 2.

**Table 2:** Basic characteristics of study participants that were hospitalized vs outpatients.

Department	TB (n = 85)			Non TB (n = 54)		
	Inpatient (n = 55)	Outpatient (n = 30)	OR (CI)	Inpatient (n = 38)	Outpatient (n = 16)	OR (CI)
Age (median–range)	36 (20–75)	35 (19–61)	0.97 (0.93–1.01)^1^	46 (20–85)	37 (22–70)	0.96 (0.91–1.00)^1^
Gender						
Male	35 (64%)	15 (50%)	0.57 (0.23–1.41)^2^	29 (76%)	7 (44%)	0.24 (0.07–0.83)^2^
Female	20 (36%)	15 (50%)	1	9 (54%)	9 (56%)	1
HIV status^3^						
HIV infected	32 (60%)	14 (54%)	1.31 (0.51–3.37)^2^	16 (43%)	9 (60%)	0.51 (0.15–1.72)^2^
HIV uninfected	21 (40%)	12 (46%)	1	21 (57%)	6 (40%)	1
Suspected site of infection categorical						
Adenitis	9 (16%)	27 (90%)	1	6 (16%)	10 (63%)	1
All other TB forms	46 (84%)	3 (10%)	46.00 (11.45–184.8)^2^	32 (84%)	6 (37%)	8.89 (2.34–33.91)^2^
Pleuritis	18 (33%)	0	NA	10 (26%)	2 (12%)	8.33 (1.34–51.67)^2^
Peritonitis	9 (16%)	1 (3%)	27.00 (2.99–245.5)^2^	12 (32%)	1 (6%)	20.00 (2.01–195)^2^
PTB with EPTB	8 (15%)	1 (3%)	24.00 (2.63–219.1)^2^	2 (5%)	0	NA
Multiorgan involvement^4^	9 (16%)	0	NA	7 (18%)	0	NA
Other^5^	2 (4%)	0	NA	1 (3%)	0	NA
Mastitis	0	1 (3%)	NA	0	3 (19%)	NA
Main comorbidities (comorbidity present %)^6^						
No comorbidity	9 (16%)	14 (47%)	1	2 (5%)	3 (19%)	1
At least one comorbidity (including HIV)	46 (84%)	16 (53%)	4.47 (1.63–12.31)^2^	26 (95%)	13 (81%)	4.15 (0.62–27.72)^2^
Hematological disease	11 (20%)	2 (7%)	3.50 (0.72–16.98)^7^	12 (32%)	1 (6%)	6.92 (0.82–58.645)^7^
Liver disease	5 (9%)	0	NA	9 (24%)	0	NA
Heavy alcohol consumption	4 (7%)	0	NA	3 (8%)	0	NA
Malignancy	6 (11%)	0	NA	13 (34%)	3 (19%)	2.25 (0.54–9.35)^7^
Renal disease	6 (11%)	0	NA	5 (13%)	1 (6%)	2.27 (0.24–21.18)^7^
Cardiovascular disease	9 (16%)	0	NA	14 (37%)	0	NA
Diabetes mellitus	0	0	NA	1 (3%)	0	NA
Other infectious diseases	11 (20%)	1 (3%)	7.25 (0.89–59.21)^7^	10 (26%)	5 (31%)	0.79 (0.22–2.83)^7^
TB complication	2 (4%)	1 (3%)	1.09 (0.10–12.59)^7^	2 (5%)	0	NA
HIV complication	13 (24%)	1 (3%)	8.98 (1.11–72.45)^7^	6 (16%)	5 (31%)	0.41 (0.11–1.62)^7^
Smoking	14 (26%)	2 (7%)	4.78 (1.01–22.69)^7^	12 (32%)	3 (19%)	2.00 (.48–8.35)^7^

Lost to follow up removed from analysis.

^1^Odds ratio per year change in age.

^2^Logistic regression. Reference given value 1.

^3^Unknown HIV status removed from analysis.

^4^Includes disseminated TB, EPTB two sites and miliary TB.

^5^Includes cyst, thyroid, ovarium, scrotum, liver, skin.

^6^Description of which comorbidities found under each heading are found in the Supplementary Table S2.

^7^Logistic regression. Not having the comorbidity used as reference.

There were significantly more non-adenitis cases among the inpatients as compared to the outpatients in both the TB (OR 46.00 (CI 11.45–184.3)) and non-TB groups (OR 8.89 (CI 2.34–33.91)), as shown in Table 2. The most common non-adenitis TB forms among TB inpatients were pleuritis (18/55, 33%), peritonitis (9/55, 16%), and multiorgan involvement (9/55, 16%). The TB patients who were recruited as inpatients had significantly more comorbidities than those recruited as outpatients, as shown in Table 2.

### Factors associated with mortality

A logistic regression model for mortality for the TB patients found that male sex and being recruited from the inpatient department were the significant factors associated with mortality (adjusted odds ratio 4.24 (CI 1.16–15.49) and 57.32 (CI 4.04–813.1)), as shown in Table 3. By exclusion of variable department (inpatient/outpatient) from the analysis, the only significant factor was not receiving ATT (adjusted OR 5.08 (CI 1.38–18.65)), also shown in Table 3.

**Table 3:** Multivariable analysis of risk factors for fatal outcome of extrapulmonary tuberculosis.

Variables in the equation	OR (CI)	aOR (CI)	aOR (CI)^1^
Categorical site of infection (non-adenitis)	5.08 (1.87–13.81)	2.66 (0.44–16.04)	0.33 (0.11–1.06)
Department (inpatient)	43.5 (5.52–343.1)	57.32 (4.04–813.1)	NA
Sex (male)	3.66 (1.39–9.59)	4.24 (1.16–15.49)	2.72 (0.89–8.28)
Age (increasing)	1.04 (1.00–1.08)	1.02 (0.96–1.08)	0.99 (0.94–1.04)
At least one comorbidity	3.16 (1.04–9.59)	2.99 (0.37–24.16)	6.29 (0.90–43.90)
HIV status (infected)	0.87 (0.35–2.17)	0.480 (0.10–2.25)	0.43 (0.10–1.82)
Not given anti-tuberculosis therapy	4.96 (1.74–14.13)	2.11 (0.51–8.78)	5.08 (1.38–18.65)

Omnibus test of model coefficients p < 0.001, Hosmer and Lemeshow test p = 0.900, Nagelkerke R square = 0.514. Model overall percentage correct: 82.3%.

^1^Analysis omitting department variable.

### Bacteriological confirmation by various diagnostic methods and mortality

**Figure 2:**
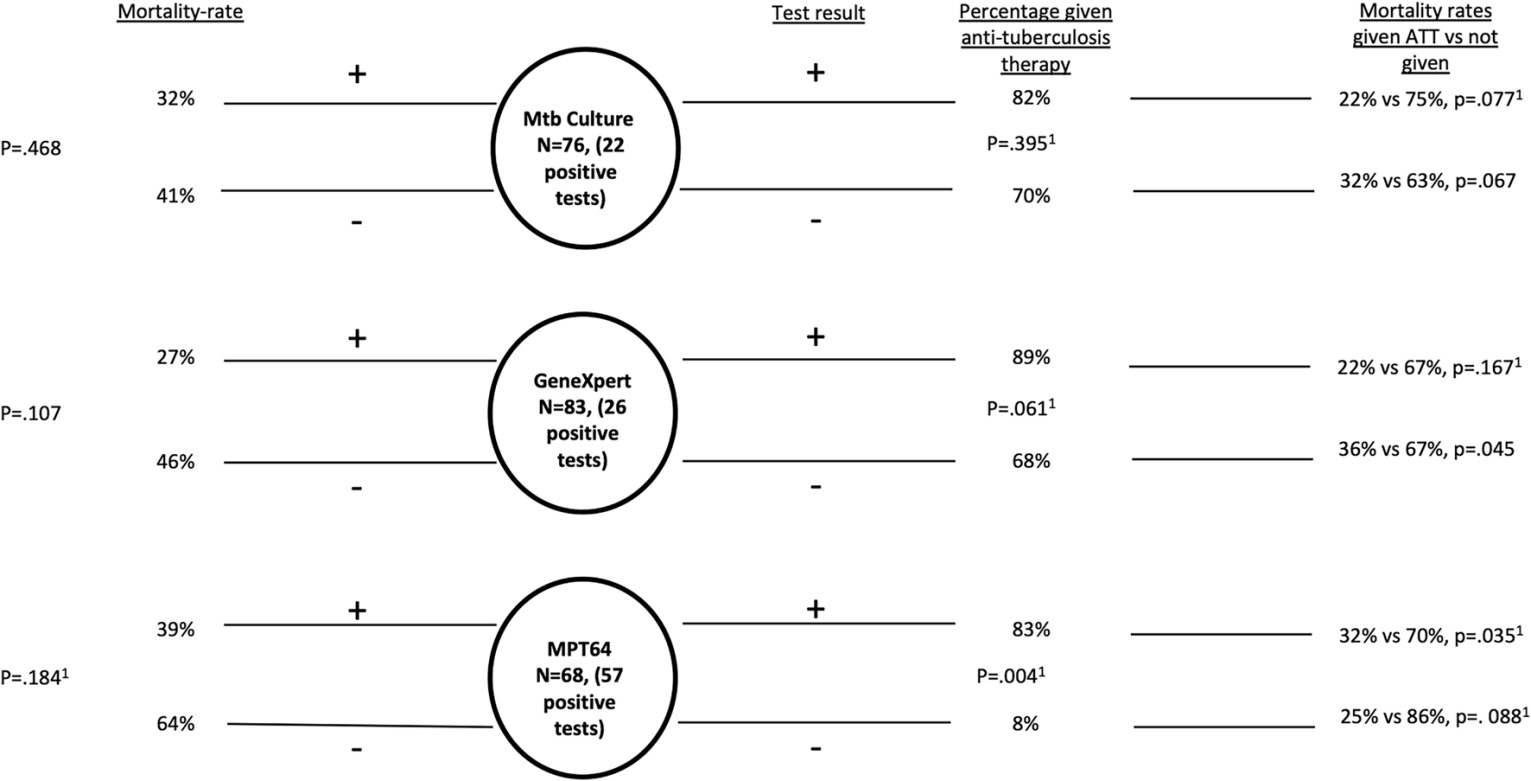
TB cases, results on diagnostic tests and mortality-rate. To the left is shown test result and correlation to mortality. The mortality rates were not different between the bacteriologically confirmed and clinically confirmed cases. To the right is shown test results, correlation to initiation of anti-tuberculosis therapy (ATT) and mortality. A negative result on the MPT64 test was less likely to lead to initiation of ATT as compared to the mycobacterial culture or GeneXpert. Initiation of ATT was associated with lesser mortality among cases confirmed by MPT64 test. Patients lost to follow up and invalid test results excluded. N = number of cases that had valid test result (positive/negative) and was not lost to follow up. ^1^Fishers exact test, other p values Chi square.

Figure 2 shows the correlation of bacteriological confirmation by culture, GeneXpert, and the MPT64 test with the initiation of ATT and mortality. The mortality rates were not different between the bacteriologically confirmed and clinically confirmed cases. A negative result on the MPT64 test was less likely to lead to initiation of ATT as compared to mycobacterial culture or GeneXpert. Initiation of ATT was associated with lesser mortality among cases confirmed by MPT64 test (mortality rate 32% vs 70%, therapy given vs not given with a positive MPT64 test, respectively, p = 0.035).

## Discussion

In our study we found a very high mortality rate of 40% and 41% among patients presenting with symptoms compatible with EPTB at Mbeya Zonal Referral Hospital regardless of TB or HIV serostatus. High inpatient mortality is critical and was probably explained by severe comorbid conditions.

A logistic regression model for the TB patients showed that being male was a significant factor contributing to mortality. Other studies on HIV infected populations have also found males to be at higher risk for mortality^14–16^.

HIV status was not significantly associated with mortality in our logistic regression model. Most individual studies have found HIV infection to be a risk-factor for EPTB^3,7^, but a recent systematic review found the heterogeneity and risk of bias to be too high to conclude^17^. The HIV infected patients in our study had a high ART coverage, but the mean current CD4 count was only 185. Viral loads were not available, and we do not have data that supports virological failure as an explanation^18^. The United Republic of Tanzania had had an estimated 71% reduction in deaths from TB in PLHIV between 2010 and 2019, and much has been attributed to the roll out of ART^19^, so wide spread virological failure is less likely.

In the logistic regression model, being recruited as an inpatient was the strongest factor associated with mortality. Unfortunately, there are few studies that focus specifically on the risk of mortality for patients presenting with symptoms compatible with EPTB in a clinical setting. Most studies on EPTB and mortality are retrospective registry studies^4,7^. There are, however, studies that try to assess the mortality risks faced by PLHIV when admitted to hospital. A major systematic review found a mortality of 20% among PLHIV admitted to hospital^20^. Studies from medical departments in hospitals in Malawi, Botswana and Tanzania have also found total inpatient adult mortality rates around 20%^16,18,21–23^, but the studies did not specifically analyze patients with a clinical suspicion of EPTB. However, Matoga et al. found a case fatality rate of TB with HIV co-infection of 40% in a Malawian hospital, and > 50% for EPTB patients with co-infection that were either newly diagnosed with HIV, naïve to or newly initiated on ART^22^. In HIV uninfected the case fatality rate for EPTB was only 13%. Our results are comparable to the subgroup of EPTB patients with HIV co-infection in this study, even though our HIV coinfected patient population was established on ART, and mortality was high regardless of HIV status. In contrast, Barak et al. found a high mortality rate of TB among the HIV uninfected (43%) as compared to the HIV infected (36%) among the inpatients at a hospital in Botswana but did not report specific numbers for EPTB^16^. Our mortality rates are also higher than those reported in retrospective EPTB studies from medical records or registries^7^.

Other explanations for the high mortality rate in our study could be that our EPTB patients had undiagnosed disseminated TB^24,25^, or that EPTB in inpatients is underdiagnosed^20,25^. Seven TB adenitis cases died in our study, 6/7 as inpatients, all having severe co-morbidities such as acquired immunodeficiency syndrome (3/7) and malignancies, which makes it likely that they had undiagnosed disseminated TB, or suspected drug-resistant TB, which in practice implies the TB was not effectively treated.

The similar mortality rates in the TB and non-TB patients, could be explained by the severe comorbidities that mimic EPTB symptoms and their high mortality rates. This is supported by a study by Kingery et al. assessing heart failure and post-discharge mortality in Tanzania, where over half of adults discharged with heart failure died within a year^26^. In addition, the successful roll out of antiretrovirals could mean that the high mortality rate of the non-TB patients is a reflection of the double burden of infectious diseases and non-communicable diseases (NCDs) that low- and middle-income countries are now facing^20,21^. Having any comorbidity and suspicion of non-adenitis was significantly associated with hospitalization. Our results imply that these NCDs serve as significant comorbidities amplifying the risk for mortality in EPTB. Unlike risk of HIV coinfection with mortality, the risk of NCDs with mortality among the EPTB patients is not well described.

When we analyzed the data by excluding the variable of department from the logistic regression model, not receiving ATT was the only significant factor associated with mortality. A quarter of the patients classified as having TB in our study did not receive ATT. In addition, 22% of the non-TB patients received ATT incorrectly. Our patients were classified based on a composite reference standard, which mimicked clinical practice in the low resource setting, where patients are often treated for presumptive EPTB without microbiological confirmation^11,12,27^. The clinicians did not, however, use our case definition when diagnosing patients, but followed national guidelines. Of the TB patients not receiving ATT, only 4/22 were confirmed by mycobacterial culture, and the rest being labelled as possible TB cases.

In our study, a negative MPT64 test result led to a less frequent initiation of ATT. The finding that fewer patients with a negative MPT64 test result received ATT could imply that clinicians had higher confidence in this new test modality and corresponds well with our previously reported high negative predictive value of the MPT64 test^11,12,27^. The poor negative predictive value of both mycobacterial culture and GeneXpert means that clinicians really cannot trust negative results. In those who had a positive MPT64 test and received ATT, we also saw a statistically significant effect on mortality. Reduced mortality rates with positive diagnostic tests are also supported by other studies and highlights the urgent need of better diagnostic tools for EPTB and ensuring that they are used^4^. Diagnostic tools for EPTB might be imperfect, but a major obstacle is also the underutilization of them, and that most patients are diagnosed with EPTB based on clinical criteria^4,5^. In 2021, only 0.2% (12/7775) of samples received in the mycobacterial culture laboratories in Tanzania were from either ascites, pleural effusions, or CSF, highlighting the lack of sampling and analysis of extrapulmonary samples^9^. In the same year, 1.2% of EPTB cases were bacteriologically confirmed nationally^28^.

In our study it is likely that a small sample size influenced our findings, and that the strong factor of hospitalization combined with a lack of data on CD4 counts/viral loads reduced the impact of HIV infection. Furthermore, no autopsies were performed, which reduces the accuracy of the final diagnosis of the patients that died^24,25^. A high number of patients being excluded due to being lost to follow up could have introduced attrition bias. As patient were classified according to a composite reference standard, there is risk of misclassification bias.

## Conclusion

We found an unacceptably high mortality rate for patients presenting with symptoms mimicking EPTB regardless of TB or HIV serostatus. Hospitalization was the main factor affecting mortality, with severe comorbidities and non-adenitis disease manifestation being risk factors for hospitalization. High mortality rates among patients that ended up being classified as non-TB patients highlights the double burden of HIV and NCDs that low- and middle-income countries are now facing. As countries are striving towards universal health coverage, focus should be on building robust health systems that can tackle both infectious diseases, such as EPTB, and NCDs such as malignancies and cardiovascular diseases.

### Supplementary Information

Supplementary Tables.

Supplementary Table S3.

## Data Availability

The data set used in this paper is available as the Supplementary Table S3.
